# Metabolism of HT-2 Toxin and T-2 Toxin in Oats

**DOI:** 10.3390/toxins8120364

**Published:** 2016-12-05

**Authors:** Jacqueline Meng-Reiterer, Christoph Bueschl, Justyna Rechthaler, Franz Berthiller, Marc Lemmens, Rainer Schuhmacher

**Affiliations:** 1 Center for Analytical Chemistry, Department of Agrobiotechnology (IFA-Tulln), University of Natural Resources and Life Sciences, Vienna (BOKU), Konrad-Lorenz-Str. 20, Tulln 3430, Austria; jacqueline.reiterer@boku.ac.at (J.M.-R.); christoph.bueschl@boku.ac.at (C.B.); franz.berthiller@boku.ac.at (F.B.); 2 Institute for Biotechnology in Plant Production, Department of Agrobiotechnology (IFA-Tulln), University of Natural Resources and Life Sciences, Vienna (BOKU), Konrad-Lorenz-Str. 20, Tulln 3430, Austria; marc.lemmens@boku.ac.at; 3 Biotechnological Processes Campus-Tulln, University of Applied Sciences, Wr. Neustadt, Konrad-Lorenz-Str. 10, Tulln 3430, Austria; justyna.rechthaler@tulln.fhwn.ac.at; 4 Christian Doppler Laboratory for Mycotoxin Metabolism, Department of Agrobiotechnology (IFA-Tulln), University of Natural Resources and Life Sciences, Vienna (BOKU), Konrad-Lorenz-Str. 20, Tulln 3430, Austria

**Keywords:** metabolomics, liquid chromatography–high-resolution mass spectrometry, stable isotopic labelling, type A trichothecenes, masked mycotoxins, xenobiotics, cereals

## Abstract

The *Fusarium* mycotoxins HT-2 toxin (HT2) and T-2 toxin (T2) are frequent contaminants in oats. These toxins, but also their plant metabolites, may contribute to toxicological effects. This work describes the use of ^13^C-assisted liquid chromatography–high-resolution mass spectrometry for the first comprehensive study on the biotransformation of HT2 and T2 in oats. Using this approach, 16 HT2 and 17 T2 metabolites were annotated including novel glycosylated and hydroxylated forms of the toxins, hydrolysis products, and conjugates with acetic acid, putative malic acid, malonic acid, and ferulic acid. Further targeted quantitative analysis was performed to study toxin metabolism over time, as well as toxin and conjugate mobility within non-treated plant tissues. As a result, HT2-3-*O*-*β*-d-glucoside was identified as the major detoxification product of both parent toxins, which was rapidly formed (to an extent of 74% in HT2-treated and 48% in T2-treated oats within one day after treatment) and further metabolised. Mobility of the parent toxins appeared to be negligible, while HT2-3-*O*-*β*-d-glucoside was partly transported (up to approximately 4%) through panicle side branches and stem. Our findings demonstrate that the presented combination of untargeted and targeted analysis is well suited for the comprehensive elucidation of mycotoxin metabolism in plants.

## 1. Introduction

The type A trichothecene mycotoxins HT-2 toxin (HT2) and T-2 toxin (T2) are secondary metabolites of *Fusarium* species such as *F. sporotrichioides*, *F. poae*, *F. armeniacum*, and *F. langsethiae* [1]. Plants, especially small grain cereals, suffer from diseases that are related to the infection with trichothecene-producing fungi, for example *Fusarium* head blight (FHB) [2]. Several recent surveys of cereals and cereal-based foods across Europe have shown that HT2 and T2 are detected most frequently and at the highest concentrations in oats and oat products [1,3,4]. In mammals, T2 is rapidly transformed into HT2 by deacetylation during digestion of infested food or feed. Both T2 and HT2 are toxic to animals and humans, affecting the immune system and causing among other things apoptosis of proliferating cells as well as inhibition of protein synthesis [5,6]. The European Food Safety Authority (EFSA) published a tolerable daily intake (TDI) of 100 ng/kg body weight for the sum of HT2 and T2 [7], while indicative levels ranging from 15 to 2000 μg/kg for cereals and cereal products were established in Recommendation 2013/165/EU from the European Commission [8].

Plants use different strategies to detoxify harmful compounds. To enhance the polarity and reactivity of xenobiotics, phase I metabolism reactions occur such as hydroxylation, hydrolysis, or oxidation. On the other hand, covalent linkage with endogenous hydrophilic molecules such as sugars, malonic acid, glutathione, and amino acids is typical for phase II metabolism. All these reactions are catalysed by enzymes, which are present within plant cells. Phase I uses esterases, amidases, and cytochrome P450-dependent hydroxylases, for example, while phase II reactions include glucosyl-, malonyl-, or glutathione S-transferases. If xenobiotics already have active functional groups, as is the case for HT2 and T2, a direct conjugation with the hydrophilic substances mentioned above can take place [9,10]. A few authors have even reported that xenobiotics or formed xenobiotic conjugates moved out of the cells again and underwent long-range transport in plants [11,12].

While mycotoxin conjugation and subsequent sequestration into cell vacuoles or cell wall biopolymers (phase III metabolism [9,10]) are generally associated with detoxification for the affected plant, these plant-derived biotransformation products can exhibit increased as well as decreased toxicity for mammals [13,14,15,16]. Moreover, the potential reactivation of detoxified derivatives in the animal intestinal tract [17] underlines the importance of plant metabolite elucidation.

Analytical methods based on liquid chromatography coupled with mass spectrometry (LC-MS) are widespread for the determination of mycotoxins and their plant metabolites. Since many plant biotransformation products of mycotoxins are not included in conventional analytical methods, also because no regulated limits exist for them in food commodities, they are called masked mycotoxins [18,19]. LC-MS/MS techniques based on multiple reaction monitoring mode are frequently used for the analysis of various (masked) mycotoxins in food products and raw materials [20,21,22]. However, much more accurate mass-to-charge (*m/z*) values are obtained by high-resolution MS (HRMS) including Orbitrap or quadrupole time-of-flight (Q-TOF) instruments. Such measurements enable a higher degree of confidence for compound identification and more structural information can be gained, especially when product ion spectra (LC-HRMS/MS) are acquired [18]. Therefore, a high-resolving instrument is highly valuable for untargeted screening of metabolites in biological matrices. The combination with stable isotopic labelling (SIL) offers to differentiate between biologically derived metabolites and unspecific signals coming from solvents, reagents, biological matrix, or instrument noise [23,24]. SIL-assisted metabolomics workflows were recently developed to study the metabolism of endogenous or exogenous tracer compounds in biological systems [25]. 

Regarding the biotransformation of HT2 and T2 in plants, a few authors have reported the occurrence of glucoside (Glc) derivatives [26,27,28,29,30,31]. McCormick et al. have investigated the anomericity of T2-Glc (*α*- or *β*-Glc) in naturally contaminated wheat and oats. Two comprehensive metabolism studies of HT2 and T2 were performed in barley [32] as well as in wheat [33]. Beyond the formation of Glc derivatives, only limited information exists about the biotransformation process of HT2/T2 in oats, although this plant was shown to be the cereal crop that is most susceptible to HT2/T2 contamination. 

The objective of this study was to elucidate the metabolism of HT2 and T2 in oats (*Avena sativa* L.) using a SIL-assisted metabolomics workflow for qualitative screening based on plant-treatment with a mixture of native and ^13^C-labelled toxin, LC-Orbitrap-MS analysis in positive and negative ion mode and automated data processing by MetExtract II software [25]. Further structural information of the formed toxin derivatives was gained by LC-HRMS/MS measurements with a LC-Q-TOF-system. Within the scope of a time course experiment, targeted quantitative analysis was performed to investigate the kinetics of metabolite formation as well as the mobility of toxin and conjugate within the plant. In this study, we elucidated novel toxin derivatives, identified and quantified the main metabolites, and gained insights into metabolic processes in oats, which might be highly valuable for risk assessment of contaminated food and feed.

## 2. Results and Discussion

### 2.1. Overview of Annotated HT2 and T2 Metabolites in Oats

For qualitative screening of plant-derived HT2 and T2 metabolites, oat panicles were treated with a 1/1 mixture of non-labelled and uniformly ^13^C-labelled toxin (termed ^12^C/^13^C sample from now on), extracted, analysed by LC-Orbitrap-MS and the data have been processed by the MetExtract II software. The automatically detected biotransformation product-derived LC-HRMS features were then manually inspected and verified, and false-positive results were removed from the data, recognisable by either implausible number of parent toxin-derived C-atoms, differences between labelled and corresponding non-labelled extracted ion chromatogram (EIC) peaks in retention time or peak shape, implausible isotope pattern, or the lack of characteristic parent toxin-derived fragments in LC-HRMS/MS spectra. In addition to automatically detected toxin derivatives, metabolites putatively missed by the strict filtering criteria of MetExtract II that had, however, been found in previous barley [32] and wheat [33] studies or as HT2 metabolites in oats, were searched for manually. Those toxin biotransformation products (M or ^12^C and respective M′ or ^13^C ion signals) showed low abundances and were thus missing isotopologs (M + 1 and/or M′ − 1) and were added to the lists when the ^12^C/^13^C signal intensity ratios were approximately 1/1 and retention times as well as accurate masses were comparable.

The resulting metabolites were annotated by the number of C-atoms derived from parent toxin. Structure units originating from the plant occurred as non-labelled moieties of the formed toxin conjugates and did not alter the ^12^C/^13^C mass shift between monoisotopic ^12^C and ^13^C-labelled metabolite ions. Further structure annotation and identification was performed on a LC-Q-TOF-system by LC-HRMS/MS measurements.

**Figure 1:**
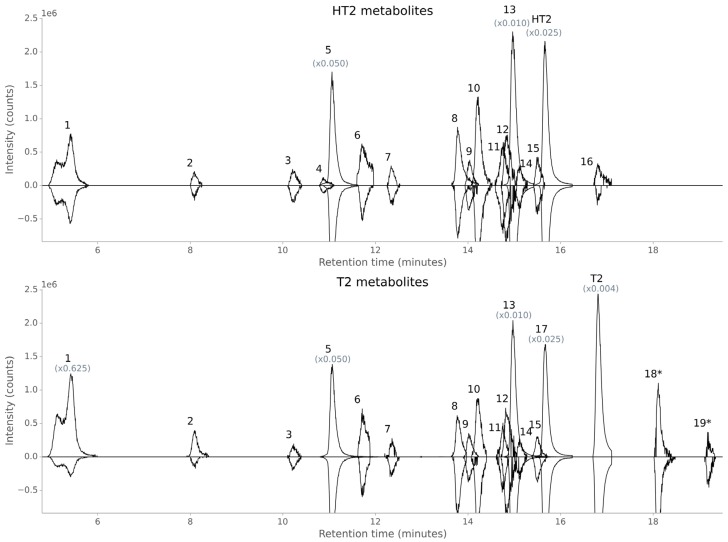
Overlay of extracted ion chromatograms (EICs) of HT2/T2 plant metabolites. (**a**) Overlaid EICs of HT2 metabolites based on HT2-treated oat sample (time point full-ripening) and Table 1; (**b**) overlaid EICs of T2 metabolites based on T2-treated oat samples (time point full-ripening and accumulated time points marked with an asterisk) and Table 2. Oat panicles were treated with a 1/1 mixture of non-labelled and uniformly ^13^C-labelled toxin, extracted and analysed by LC-Orbitrap-MS in positive and negative ion mode and MetExtract II software. Non-labelled metabolite form is depicted with positive intensity (up) and corresponding ^13^C-labelled metabolite form with negative intensity (down). HT2, HT-2 toxin; T2, T-2 toxin.

A total of 16 HT2 and 17 T2 metabolites were found in oat samples (Table 1 and Table 2 and Figure 1), which were present as different ion species (mainly [M+NH_4_]^+^, [M+Na]^+^, [M+H]^+^, [M+K]^+^ and [M+HCOO]^−^, [M−H]^−^, [M+Cl]^−^) or in-source fragments.

In good agreement with previous studies on the metabolism of HT2 and T2 in barley [32] and wheat [33], most T2 metabolites matched those found for HT2 also in oats due to the rapid loss of the acetyl group (2 C-atoms) at C-4 position of T2. Only two metabolites were detected with intact T2 (24 C-atoms) backbone, namely 3-acetyl-T2 (18) and feruloyl-T2 (19). Taking HT2 (22 C-atoms) as a starting point, losses of the isovaleryl group (5 C-atoms) or of the acetyl group (2 C-atoms) were observed *in planta* (metabolites 1, 2, and 8). In agreement with that, Cole [9] reported that hydrolysation of xenobiotic esters is a common phase I metabolism route. 

All metabolites showed ^12^C/^13^C signal intensity ratios of approximately 1/1, except for the T2 metabolites 15-acetyl-T2-tetraol-Glc (1) and dehydro-15-acetyl-T2-tetraol-Glc (2). These two substances were not recognised by the untargeted approach because of unexpected ^12^C/^13^C signal intensity ratios (2/1 and higher). Similar to the previous barley study [32], a minor impurity of non-labelled neosolaniol in the ^12^C/^13^C-T2 treatment solution has probably led to higher ^12^C/^13^C signal intensity ratios of the mentioned metabolites due to a parallel metabolism process of neosolaniol in the treated plants. 

As can be seen from the toxin metabolites in Table 1 and Table 2 and Figure 1, hydroxylation and glucosylation metabolism processes dominated in oats. These findings are concordant with Cole [9] who described that xenobiotics are often hydroxylated by cytochrome P450-dependent hydroxylases and glucosylated by *O*-glucosyltransferases in plants. While the glucosylation of HT2, forming a *β*-linked d-glucose conjugate (confirmed by comparison with respective standard), was an important detoxification strategy in oats, no T2-Glc was detected; it was, however, found in barley [32], as well as in naturally contaminated wheat and oats [31]. Additionally, in contrast to previous xenobiotic metabolism studies [32,33] and [15,34,35] hardly any malonic acid conjugations were detected in oats, except the less abundant HT2-malonylglucoside (HT2-MalGlc (14)), which was close to the analytical limit of detection (LOD, signal-to-noise ratio of 3 in matrix) (see Figure 1). Thus, it is assumed that the quantity or activity of HT2/T2 metabolising *O*-malonyltransferases is lower in oats than in barley and wheat under the tested conditions.

Interestingly, in the present study six novel HT2/T2 metabolites were detected for the first time in plants; these were putatively annotated as hydroxy-HT2-hexosylglucoside (hydroxy-HT2-HexGlc (two isomers 3 and 4)), hydroxy-HT2 (6), hydroxy-HT2-anhydro-HexGlc (7), HT2-malylGlc (12) and HT2-anhydro-HexGlc (15). While five of them seem to be closely related to already known HT2/T2 metabolites, HT2-malylGlc was presumably formed by the covalent binding of malic acid to a glucose moiety of the initially formed HT2-3-*O*-*β*-d-Glc. The conjugation with organic acids is widely known in plant metabolism. Abdel-Farid et al. [36], for example, described malate conjugates of cinnamic acid derivatives in *Brassica rapa* leaves and Fujisawa et al. [37] elucidated a malate conjugate of a pesticide in leaf cell suspension of *Brassica oleracea*.

Our data indicate that not only is there a deacetylation reaction from T2 into HT2, but also the reverse reaction from HT2 to T2 occurs in plants, although to a much lower extent (see Table 1 and Figure 1). While in wheat C-3 and C-4 acetylated HT2 (4-acetyl-HT2 is corresponding to T2) were probably detected [33], the present study revealed solely the putative C-4 acetylated HT2 with a ^12^C/^13^C mass shift of Δ22.074 u (theoretical value). It is worth mentioning that an impurity of ^13^C-T2 in the ^12^C/^13^C-HT2 treatment solution was also observed with the M′ − 2 isotopolog having the same *m/z* value as the labelled metabolite form of acetylated HT2. However, a significant increase of the M′ − 2 isotopolog of ^13^C-T2 was recognised in the respective MS spectra and the metabolite ^12^C/^13^C signal intensity ratio was approximately 1/1, which confirmed that this toxin derivative was T2, formed by HT2.

### 2.2. Structure Elucidation by LC-HRMS/MS

As a starting point of structure annotation of detected HT2 and T2 metabolites, TracExtract output of LC-HRMS measurements provided the number of parent toxin-derived C-atoms and the assigned ion species of intact toxin derivatives (Table 1 and Table 2). Further metabolite information was obtained by LC-HRMS/MS spectra acquired with a LC-Q-TOF-system.

All metabolites for which LC-HRMS/MS spectra could be acquired in the positive ion mode showed the same characteristic HT2 or T2 fragments as in [32]. Spectra were additionally checked for typical fragment ions of hexose (such as glucose) and malonylglucose moieties. The relative mass deviations between predicted, and measured precursor or fragment ions did not exceed 22 ppm. 

#### 2.2.1. Confirmation of Previously Found HT2 and T2 Metabolites

HT2, T2, HT2-3-*O*-*β*-d-Glc, and 3-acetyl-T2 were identified by the comparison of retention times (using additionally longer gradient method 3 from study [32]), accurate masses, and LC-HRMS/MS spectra with authentic standards. In the same way, the metabolites (1, 2, 5, 8–11, 14, 19) were confirmed by comparison with those known from previous barley [32] and wheat [33] studies.

As a result, the metabolites (1, 2, 5, 8–11, 13, 14, 16–19) were shown to be identical to those from the studies with barley and wheat. An LC-HRMS/MS spectrum in negative ion mode of T2-triol-Glc was additionally annotated and is illustrated in Supplementary Figure S1. The abundance of dehydro-15-acetyl-T2-tetraol-Glc (2) was too low to generate meaningful product ion spectra.

#### 2.2.2. Elucidation of Novel HT2 and T2 Metabolites

Moreover, the structure of novel oat-derived HT2/T2 metabolites was elucidated by the comparative interpretation of LC-HRMS/MS spectra of native and corresponding ^13^C-labelled metabolites, acquired in the same LC-HRMS/MS run. Within overlaid ^12^C/^13^C LC-HRMS/MS spectra, fragment ions containing a part of the parent toxins showed ^12^C/^13^C mass shifts proportional to the parent toxin-derived number of C-atoms. On the other hand, for fragment ions of conjugated moieties originating from the native plant, no ^12^C/^13^C mass shifts were observed. The product ion spectra were interpreted and calculated sum formulas as well as ^12^C/^13^C mass shifts partially confirmed by the help of the FragExtract module of the MetExtract II software. Additionally, the relative elution order of the putative toxin derivatives was checked for plausibility. 

The six HT2/T2 metabolites (3, 4, 6, 7, 12, and 15), which are described here for the first time contained intact HT2 carbon skeleton with a ^12^C/^13^C mass shift of Δ22.074 u (theoretical value). LC-HRMS/MS spectra of these novel metabolites are illustrated in Supplementary Figures S2–S6. The positive and negative ion mode derived LC-HRMS/MS spectra of (3/4, 7, 12, and 15) suggest that at least one hexose (most probably glucose) molecule is conjugated to HT2 showing the typical fragment ions of a hexose moiety, namely *m/z* 145.0495, *m/z* 127.0390, and *m/z* 161.0455, corresponding to [hexose–2 H_2_O+H]^+^, [hexose–3 H_2_O+H]^+^ and [hexose–H_2_O–H]^−^, respectively. 

Two putative hydroxy-HT2-HexGlc isomers (3) and (4) were detected with the same *m/z* values at different retention times. Since product ion spectra (Figure S2) were similar to those of hydroxy-HT2-Glc (5) and HT2-HexGlc (10, 11) respectively, it is assumed that (3) and (4) were formed by the hydroxylation of the two isomers of (10) and (11). The loss of the additional oxygen atom always coincided with the loss of isovaleric acid (C_5_H_10_O_2_, ∆102.0681 u) such as *m/z* 647.2540 [M–O–isoval acid+H]^+^ and *m/z* 485.1987 [M–O–isoval acid–hexoside+H]^+^, suggesting that the hydroxyl group is linked to the isovaleryl group. Moreover, after cleavage of two hexoside moieties corresponding to two times Δ162.053 u (theoretical value) from the [M+H]^+^ ion, *m/z* 441.2093 [hydroxy-HT2+H]^+^ was left.

Hydroxy-HT2 (6) was annotated by the detection of an intact HT2 backbone and a mass shift of Δ15.994 u between *m/z* 441.2105 [M+H]^+^ and theoretical *m/z* 425.2170 [HT2+H]^+^ (Figure S3). Two losses of water resulting in *m/z* 423.1968 [M–H_2_O+H]^+^ and *m/z* 405.1867 [M–2 H_2_O+H]^+^ were observed. Moreover, the product ion spectra of (6) showed the simultaneous losses of the hydroxy–oxygen atom together with isovaleric acid, indicating again that the location of the hydroxyl group was at the isovaleryl group of HT2.

For putative HT2-malylGlc (12) the intact HT2 backbone and fragment ions corresponding to a conjugated hexose (most probably glucose) molecule were confirmed (Figure S4). Subtracting the theoretical *m/z* 587.2698 [HT2-Glc+H]^+^ from *m/z* 703.2830 [M+H]^+^ gave Δ116.013 u in accordance with malic acid minus water. The malylglucose moiety was easily cleaved off from the intact toxin derivative (12) by LC-HRMS/MS resulting in *m/z* 425.2157 [HT2+H]^+^, *m/z* 407.2055 [HT2–H_2_O+H]^+^ and losses of water from malylglucose, namely *m/z* 279.0689 [malylglucose–H_2_O+H]^+^ and *m/z* 261.0604 [malylglucose–2 H_2_O+H]^+^. Typical fragments of malic acid reported in the PubChem database [38] were also detected such as *m/z* 135.0260 [malic acid+H]^+^ and *m/z* 89.0234 [malic acid–formic acid+H]^+^ in positive ion mode or *m/z* 115.0037 [malic acid–H_2_O–H]^−^ and *m/z* 71.0141 [malic acid–H_2_O–CO_2_–H]^−^ (*m/z* 71.014 also detected in LC-HRMS/MS spectrum of HT2-3-*O*-*β*-d-Glc originating from glucose moiety) in negative ion mode (data not shown).

Two of the detected toxin derivatives are assumed to carry an anhydro-hexose moiety corresponding to hexose minus water. The tentative hydroxy-HT2-anhydro-HexGlc (7) and HT2-anhydro-HexGlc (15) were closely related, since the fragmentation patterns were very similar (Figures S5 and S6). Most fragment ions in positive ion mode LC-HRMS/MS spectra had identical *m/z* values. This can be explained by the simultaneous loss of the additional hydroxy–oxygen atom with each loss of isovaleric acid of (7) resulting in *m/z* 629.2390 [(7)–O–isoval acid+H]^+^ and *m/z* 569.2193 [(7)–O–isoval acid–acetic acid+H]^+^, for example, which were identical to *m/z* 629.2359 [(15)–isoval acid+H]^+^ and *m/z* 569.2205 [(15)–isoval acid–acetic acid+H]^+^. While for (15), *m/z* 425.2158 [HT2+H]^+^ was observed, (7) has solely shown *m/z* 423.1992 [hydroxy-HT2–H_2_O+H]^+^. Typical fragment ions of hexose moieties were detected as *m/z* 145.0488/145.0490 [hexose–2 H_2_O+H]^+^, *m/z* 127.0375/127.0374 [hexose–3 H_2_O+H]^+^ and even *m/z* 163.0583/163.0590 [hexose–H_2_O+H]^+^. In contrast to all other HT2-Glc derivatives, the hexose-related fragment ions showed higher relative abundances in the LC-HRMS/MS spectra of (7) and (15). Moreover, product ions of putative dihexosides such as *m/z* 307.0993/307.1007 [dihexoside–2 H_2_O+H]^+^, *m/z* 289.0902/289.0890 [dihexoside–3 H_2_O+H]^+^, *m/z* 271.0803/271.0784 [dihexoside–4 H_2_O+H]^+^, *m/z* 253.0670/253.0696 [dihexoside–5 H_2_O+H]^+^, *m/z* 207.0645/207.0623 [dihexoside–6 H_2_O–CO+H]^+^, and *m/z* 177.0543/177.0551 [dihexoside–6 H_2_O–CO–CH_2_O+H]^+^ were present with high relative intensities. Interestingly, some of these fragment ions were also found in positive ion mode product ion spectra of the two isomers HT2-HexGlc (10 and 11), however with very low abundances. This indicates that both isomers include a dihexoside, rather than two single sugar moieties that are linked to the C-3 and C-4 position of HT2. Hedin and Phillips [39] reported the structure elucidation of different sugars and related derivatives with chemical ionisation mass spectrometry. They described the fragmentation pattern of glucose (and other aldohexoses) to contain fragment ions at *m/z* 163, 145, and 127 as well as of dihexosides, showing, amongst others, *m/z* 307 and 289. Since in negative ion mode LC-HRMS/MS spectra (data not shown) of (15) *m/z* 585.2525 [HT2-Glc–H]^−^ and of (7) the respective *m/z* 601.2484 [hydroxy-HT2-Glc–H]^−^ were detected, our results suggest that the second conjugated hexose moiety was modified in the plant by the cleavage of one water molecule. This has probably led to more dominant further losses of water in positive ion mode LC-HRMS/MS spectra and thus higher relative intensities of respective fragment ions. Taken together, we conclude that (7) and (15) were most probably derived from one isomer of HT2-HexGlc and that (7) was presumably formed by the hydroxylation of the HT2 isovaleryl group of metabolite (15). Another plausible metabolic pathway is the formation of (7) by the cleavage of one hexose–water molecule from intermediate hydroxy-HT2-HexGlc (3/4).

### 2.3. Kinetics of Metabolite Formation and Distribution

Separate oat panicles were treated with 200 μg non-labelled HT2 (corresponding to 0.47 µmol) and T2 (corresponding to 0.43 µmol) (stated ^12^C samples) in five biological replicates and harvested immediately (zero), after one, three, or seven days, or at the full-ripening stage (time course experiment). Plants were cut into three parts, namely treated spikelets, non-treated spikelets, and pooled stem plus side branches, each of which was analysed separately on a LC-Q-TOF-MS-system in full scan mode.

#### 2.3.1. Absolute Quantification 

Since standards were available, parent toxins plus HT2-3-*O*-*β*-d-Glc were quantified in treated spikelets. The absolute amounts were calculated in μmol/treated part and put in relation to the amounts of recovered HT2 and T2 at time point zero days. All measured concentration values were corrected by the respective standard purities and matrix effects, which were between 73% and 86% in 400-fold diluted oat extracts.

**Figure 2:**
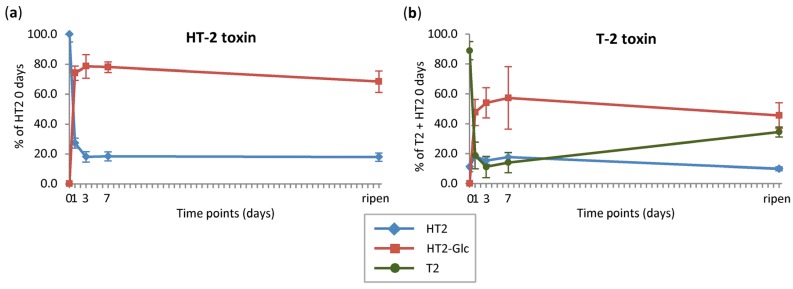
Time courses of native toxins and major plant-derived metabolites. (**a**) Time course of HT2 (HT-2 toxin) metabolism; (**b**) time course of T2 (T-2 toxin) metabolism. Oat panicles were treated with 200 μg non-labelled HT2 and T2 and harvested directly (zero), and one, three, or seven days after treatment, or at full-ripening stage (for each time point toxins were applied on separate panicles in five biological replicates). Quantification was performed on a LC-Q-TOF-system; absolute amounts were calculated in μmol/treated part and put in relation to the amounts of recovered HT2 and T2 at time point zero days (percent mean value ± standard deviation is illustrated). HT2-Glc, HT2-3-*O*-*β*-d-glucoside.

Figure 2 shows the kinetics of HT2/T2 metabolism. Since the harvesting procedure for each oat panicle took approximately 5–10 min until freezing in liquid nitrogen and T2 was rapidly converted to HT2, at time point zero 0.05 ± 0.01 μmol HT2, corresponding to approximately 11% of the added T2, were already found in T2-treated oats. Therefore, the initially formed HT2 amount was added to that of T2 at time point zero. The recoveries of parent toxins in oats immediately harvested after treatment were 90% (0.42 ± 0.02 μmol) for HT2 and 99% (0.43 ± 0.02 μmol) for T2 plus HT2.

As depicted in Figure 2, parent toxins HT2 and T2 were metabolised very fast in oats. After one day, only 27% HT2 (0.12 ± 0.01 μmol in HT2-treated oats) and 19% T2 (0.08 ± 0.04 μmol in T2-treated oats) were left. However, in T2-treated oats an increase in parent toxin T2 to 35% (0.15 ± 0.01 μmol) was observed until the full-ripening stage. HT2-3-*O*-*β*-d-Glc was the main metabolite present with 74% (0.31 ± 0.02 μmol) in HT2-treated oats and 48% (0.20 ± 0.04 μmol) in T2-treated oats after one day. In comparison with the previous barley [32] and wheat [33] studies, oats was the plant species with the highest turnover from HT2 and T2 into HT2-3-*O*-*β*-d-Glc. Over time (time points three and seven days), the amounts of HT2-3-*O*-*β*-d-Glc showed a slight increase but dropped subsequently until the full-ripening point. The later decrease indicates that other HT2/T2 biotransformation products were formed by further metabolic reactions of HT2-3-*O*-*β*-d-Glc. As the sum of parent toxins plus these absolutely quantified metabolites made up between 86% and 101% in HT2-treated plant parts and between 80% and 100% in T2-treated plant parts, a maximum of 14% or 20% was left. This remaining proportion includes other HT2/T2 biotransformation products and/or not-recognised amounts of toxin derivatives due to transport in non-treated plant parts (for example non-treated spikelets, stem, side branches, leaves, and roots) or incorporation into the plant matrix.

#### 2.3.2. Relative Quantification 

**Figure 3:**
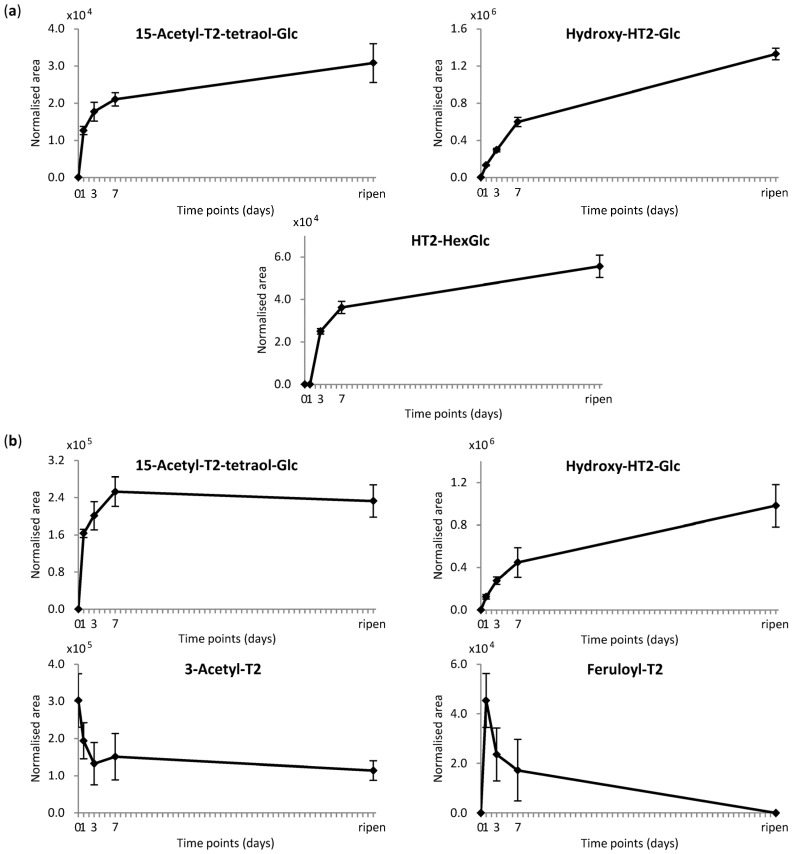
Relative time courses of plant-derived metabolites. (**a**) Relative time courses of HT2 (HT-2 toxin) metabolites; (**b**) relative time courses of T2 (T-2 toxin) metabolites. Oat panicles were treated with 200 μg non-labelled HT2 and T2 and harvested directly (zero), and one, three, or seven days after treatment, or at the full-ripening stage (for each time point toxins were applied on separate panicles in five biological replicates). Relative quantification was performed on a LC-Q-TOF-system; peak areas of ammonium adducts were normalised by respective weight of treated plant part (area mean value ± standard deviation is illustrated). Glc, glucoside; HexGlc, hexosylglucoside.

Relative quantification of other HT2/T2 metabolites in treated plant parts is depicted in Figure 3. Only for three HT2 and four T2 metabolites were the abundances over time high enough to create meaningful time courses. The formation of T2 in ^12^C-HT2-treated oat samples was not depicted, since no differentiation could be made between T2 contamination and T2 originating from parent toxin HT2.

**Figure 4:**
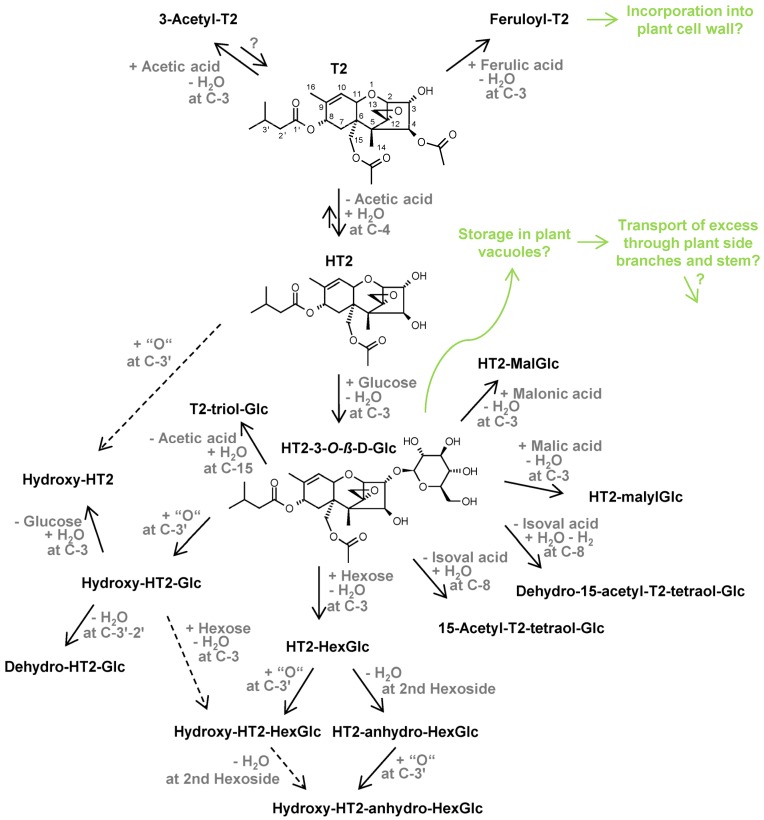
Proposed metabolic fate of HT2 (HT-2 toxin) and T2 (T-2 toxin) in oats. Analysis was performed by liquid chromatography–high-resolution mass spectrometry (LC-HRMS). Structure annotation was based on accurate masses, number of parent toxin-derived C-atoms, LC-HRMS/MS spectra, and on assessment of retention times. Structure identification was based on comparisons with available standards. Glc, glucoside; MalGlc, malonylglucoside; HexGlc, hexosylglucoside; isoval acid, isovaleric acid.

Figure 3 shows that most HT2/T2 metabolites increased in concentration until the full-ripening point. The same applies to metabolites hydroxy-HT2-HexGlc (3, 4), hydroxy-HT2-anhydro-HexGlc (7), T2-triol-Glc (8), dehydro-HT2-Glc (9), HT2-HexGlc (10, 11, also in T2-treated plants) and HT2-MalGlc (14). Additionally, metabolites dehydro-15-acetyl-T2-tetraol-Glc (2), hydroxy-HT2 (6), HT2-malylGlc (12), and HT2-anhydro-HexGlc (15) were only obviously detected in ^12^C/^13^C samples harvested after ripening and measured with LC-Orbitrap-MS due to a lower injection volume of the ^12^C samples applied on LC-Q-TOF-MS and different instrument sensitivities. These late maxima suggest again that they are derived from the rapidly formed main metabolic intermediate HT2-3-*O*-*β*-d-Glc. Figure 4 depicts the proposed metabolic fate of HT2 and T2 in oats based on our findings. For both parent toxins the central metabolic pathway was congruent, including the glucosylation of HT2 at the C-3 position, while for T2 the C-4 acetyl group was firstly cleaved to form HT2. Taking HT2-3-*O*-*β*-d-Glc as a starting point, hydrolytic cleavages of ester groups as well as further covalent linkages with different polar moieties were observed with highest amounts after ripening. 15-Acetyl-T2-tetraol-Glc (1), dehydro-15-acetyl-T2-tetraol-Glc (2), and T2-triol-Glc (8) resulted from the hydrolytic cleavages of the isovaleryl group at C-8 position or the acetyl group at C-15 position, respectively. After HT2-3-*O*-*β*-d-Glc formation, putative malonic acid (14) or malic acid (12) was conjugated to the glucoside. HT2-HexGlc (10, 11) was most probably formed by the extension of the glucoside with a second hexose molecule. The subsequent formation of tentative HT2-anhydro-HexGlc (15) resulted from the loss of one water molecule, presumably from one of the hexoside residues. Moreover, many metabolites were formed by hydroxylation of the C-8 isovaleryl group such as hydroxy-HT2-Glc (5), hydroxy-HT2-HexGlc (3, 4), hydroxy-HT2-anhydro-HexGlc (7), and hydroxy-HT2 (6). Putative dehydro-HT2-Glc (9) resulted most probably from the cleavage of the additional hydroxyl group of hydroxy-HT2-Glc (5), corresponding to a loss of one water molecule. 

While it can be hypothesised that hydroxy-HT2 (6) followed another metabolic route, formed directly by the hydroxylation of unmodified HT2, its maximum abundance at the latest time point (full ripening) indicates that HT2-3-*O*-*β*-d-Glc was also transformed into hydroxy-HT2-Glc and then the Glc moiety was cleaved off (see Figure 4). This is in agreement with the observation that the hydroxylation reaction occurred more slowly and to a lesser extent than the glucosylation of the parent toxins, as can be seen in Figure 1, Figure 2 and Figure 3 when comparing hydroxy-HT2-Glc (5) with HT2-3-*O*-*β*-d-Glc (13) and hydroxy-HT2-HexGlc (3, 4) with HT2-HexGlc (10, 11). Taken together, both typical phase I and II metabolic transformations were observed in oats, while phase II metabolism appeared to be faster and more dominant.

Not surprisingly, the time courses of 15-acetyl-T2-tetraol-Glc differed in HT2- and T2-treated oats (Figure 3). The abundances were approximately 10 times higher in T2-treated oats and the concentration maxima arose at different time points. This can be explained by the small impurity of non-labelled neosolaniol in the ^12^C-T2 treatment solution, which might be additionally converted to 15-acetyl-T2-tetraol-Glc, but probably faster than T2. 

Interestingly, metabolites including the intact T2 skeleton as 3-acetyl-T2 and feruloyl-T2 showed contrary kinetics of formation. The maximum abundance of 3-acetyl-T2 was already reached at zero days and quantification revealed an amount of <0.5% relative to the amounts of recovered T2 plus HT2. Feruloyl-T2 was at its highest concentration one day after treatment. Thus, similar to former barley [32] and wheat [33] studies, a second metabolic pathway was observed for T2 being modified by the covalent binding of acetic acid and putative ferulic acid before the C-4 acetyl group has been hydrolysed to form HT2 (see Figure 4). Moreover, Figure 3 shows that with increasing time the amounts of 3-acetyl-T2 and feruloyl-T2 decreased. Since the hydrolysis of acetyl groups is a common metabolic process monitored in this study, it is assumed that the reverse reaction from 3-acetyl-T2 to T2 could be responsible for that decrease. This hypothesis additionally correlates well with the observed increase of T2 between seven days after treatment and the full-ripening time point (Figure 2). Although the maximum of the T2 formation from parent toxin HT2 (2.1) could not be determined, both reverse reactions from 3-acetyl-T2 and HT2 into T2 have probably led to a rise in the amount of T2. For the decrease of feruloyl-T2 after one day, we presume continuous incorporation into the plant cell wall. This is in agreement with Iiyama et al. [40], who have reported that phenolic acids such as ferulic acid are involved in cell wall strengthening of plants, being a significant strategy to reduce the access of pathogens. Additionally, McKeehen et al. [41] have described the involvement of ferulic acid in resistance mechanisms of grains against *Fusarium* species. 

#### 2.3.3. Mobility of Parent Toxins and HT2-3-*O*-*β*-d-Glc in Oats

Semi-quantitative analysis of HT2, T2, and HT2-3-*O*-*β*-d-Glc in non-treated plant parts (non-treated spikelets and pooled stem plus side branches) was performed and amounts were related to the amounts of recovered HT2 and T2 in treated plant parts at zero days. 

While HT2 was below the LOD in any of the HT2- and T2-treated oat samples, T2 was found to be <0.2% in non-treated plant parts (T2-treated oats). This difference may well be due to a higher analytical sensitivity for T2 compared with HT2 rather than a different mobility behaviour of the two toxins. As Table 3 and Table 4 show, solely HT2-3-*O*-*β*-d-Glc was detected, with considerable amounts higher than 1%.

Our results demonstrate that the main metabolite HT2-3-*O*-*β*-d-Glc was slightly mobile in oat plants, while the less polar parent toxins were hardly transported. As can be seen in Table 3 and Table 4, low proportions of HT2-3-*O*-*β*-d-Glc moved through the stem and side branches but did not end up in the adjacent non-treated spikelets. The maximum was reached at one day, with approximately 4% in HT2-treated and 1% in T2-treated oats, and a decrease was observed over time until the full-ripening stage.

According to Coleman et al. [10], it can be hypothesised that the xenobiotics HT2 and T2 passively diffused into the plant cells of treated spikelets, landing in cytosol, where many enzymes such as *O*-glucosyltransferases were responsible for the metabolic reactions. As the main metabolic process, forming HT2-3-*O*-*β*-d-Glc, occurred very fast and with high conversion rates in treated spikelets, it is suggested that these parts of oat underwent a saturation process with HT2-3-*O*-*β*-d-Glc. Although the storage of conjugates in cell vacuoles is an important mechanism for xenobiotics disposal [9], we assume that the local capacity for HT2-3-*O*-*β*-d-Glc storage was temporarily exhausted and the plants consequently transported the excess through side branches and stem towards roots (see Figure 4). This movement of HT2-3-*O*-*β*-d-Glc was probably conducted via the phloem, which is known to act as a transport system for information and nutrients from source to the sink tissue [42]. As in general the uptake of sugars into the phloem is mediated by carrier proteins, we assume that our main toxin derivative was either recognised by such a carrier or moved passively (diffusion) through the cell walls and membranes. Moreover, the observed decrease of HT2-3-*O*-*β*-d-Glc in the pooled stem plus side branches over time was probably due to the further transport to lower, not investigated plant parts, further metabolic transformation, the incorporation into the plant matrix or the further storage in plant vacuoles, which are distributed over the whole oat plant. 

Adding the estimated values of HT2-3-*O*-*β*-d-Glc in non-treated plant parts to the total amounts of quantified HT2, T2, and HT2-3-*O*-*β*-d-Glc (2.3.1) in treated plant parts over time led to a maximum remaining quantity of approximately 13% in HT2-treated and 19% in T2-treated oats.

## 3. Conclusions 

The application of an untargeted analytical approach based on isotopic labelling, LC-HRMS, and LC-HRMS/MS measurements revealed 16 HT2 and 17 T2 metabolites in oats. Many related derivatives were formed by typical phase I and II metabolic processes, including hydrolysis of ester groups, glycosylation, and hydroxylation reactions (Figure 4). Six of the detected biotransformation products are described for the first time in plants, namely two isomers of putative hydroxy-HT2-HexGlc as well as tentative hydroxy-HT2, hydroxy-HT2-anhydro-HexGlc, HT2-malylGlc, and HT2-anhydro-HexGlc. In contrast to HT2/T2 metabolism in barley [32] and wheat [33], malonylation played only a minor role in oats.

After time course experiments, HT2-3-*O*-*β*-d-Glc has been found to be the main metabolic intermediate of both parent toxins HT2 and T2, which was rapidly formed and further metabolised. Under the tested conditions, the sum of HT2, T2, and HT2-3-*O*-*β*-d-Glc amounted to between 88% and 106% in HT2-treated oats and between 81% and 100% in T2-treated oats, leading to a maximum remaining proportion of approximately 13% and 19%, respectively, for all other derivatives including those that have not been captured here. Relative time courses of other metabolites showed that most of them were derived from HT2-3-*O*-*β*-d-Glc, except 3-acetyl-T2 and feruloyl-T2, which followed a separate metabolic pathway. Interestingly, semi-quantitative analysis of HT2, T2, and HT2-3-*O*-*β*-d-Glc of non-treated plant parts revealed that low fractions of HT2-3-*O*-*β*-d-Glc were mobile in oats and probably transported by the phloem, while the movement of parent toxins was negligible. The elucidation of HT2/T2 metabolism in plants might be an important contribution to the risk assessment of contaminated cereals. However, further studies are needed to determine the toxicity of the detected metabolites, including the health hazards associated with potential reactivation by their hydrolysis in the intestinal tract of mammals.

## 4. Materials and Methods

### 4.1. Chemicals and Reagents

Methanol and acetonitrile (HPLC grade) were obtained from VWR (Vienna, Austria), ammonium formate solution (5 M) was provided by Agilent Technologies (Waldbronn, Germany), and formic acid (LC-MS grade) was purchased from Sigma-Aldrich (Vienna, Austria). Water was successively purified by reverse osmosis and an ELGA Purelab Ultra Mk2 Analytic system from Veolia (Vienna, Austria). For plant treatment, crystalline standards of non-labelled as well as uniformly ^13^C-labelled (degree of enrichment between 99.3 atom% and 99.6 atom% ^13^C) HT2 and T2 (purity between 85% and 99%), were purchased from Romer Labs (Tulln, Austria). Oat treatment solutions of HT2 and T2 for qualitative screening of plant-derived toxin derivatives consisted of a 1/1 (*v*/*v*) mixture of non-labelled and labelled toxin (1000 mg/L per toxin), whilst for time course experiments, solutions of non-labelled toxin with 2000 mg/L were prepared. Besides low amounts of non-labelled neosolaniol, none of the known type A trichothecenes were present in treatment solutions as impurities over 1%. The solvent for all treatment solutions was acetonitrile/water 1/1 (*v*/*v*), which additionally served as a blank treatment solution (mock). HT2 and T2 standards for quantification purposes were obtained from Romer Labs as stock solutions of 100 mg/L and 101 mg/L, respectively, in acetonitrile. The analytical standards HT2-3-*O*-*β*-d-Glc and 3-acetyl-T2 (purity ≥ 95%) were enzymatically or chemically synthesised, characterised by nuclear magnetic resonance measurements (unpublished data), and dissolved in acetonitrile to obtain concentrations of 1000 and 5000 mg/L, respectively.

### 4.2. Plant Cultivation

The oat (*Avena sativa* L.) variety “Eneko”, originating from a cross between “Triton” × “Flaemingsprofi”, was selected for the experiments. This is a commercial spring oat variety bred by Saatzucht Edelhof in Austria [43]. Seeds of “Eneko” were germinated in pots filled with a substrate as described by Meng-Reiterer et al. [32]. In each pot five seedlings were planted. During the whole experiment the pots were watered when required. Plants were germinated in the greenhouse and after germination transferred to a growth chamber. At the end of tillering, 2 g of a mineral fertiliser (COMPO Blaukorn ENTEC, Muenster, Germany; N/P/K/Mg: 14/7/17/2) was applied per pot. Settings for light, temperature, and relative air humidity in the growth chamber were computer-controlled. Light intensity was 560 μmol·s^−1^·m^−2^ at 1 m above the soil and relative air humidity was set between 60% and 70% during plant growth. Temperature (day/night) and duration of illumination (hours) varied according to the development stage of the plants (for details, see [32]). From the start of flowering until the end of the experiments, including application of the treatment solutions and sampling, settings were 20 °C/18 °C (day/night), 16 h light and 60%–70% relative air humidity.

### 4.3. Treatment and Sampling of Oat Plants

Oat panicles at flowering stage (spikelets in the middle of panicle flowered) were randomly selected for the three treatment groups HT2, T2 and mock. For each treatment variant, 20 spikelets in the upper area of the panicle were labelled with wool threads and each spikelet was treated with 5 μL of the respective treatment solutions with an electronic pipette, starting with the highest ones. Thus, an amount of 100 μg of non-labelled and 100 μg of labelled toxin per panicle was applied for qualitative screening experiment or 200 μg of non-labelled toxin per panicle for time course experiments. After each treatment step, small transparent plastic bags were sprayed with purified water, put over the panicles to prevent drying and promote diffusion of the treatment solutions into the plant cells, and removed 24 h (±2 h) later. On the day of harvest, treated oat panicles were cut into three parts, which were the 20 treated spikelets, the remaining non-treated spikelets, and pooled stem plus side branches. After weighing, the plant parts were rapidly frozen in liquid nitrogen and stored at −80 °C until further analysis.

#### 4.3.1. Qualitative Screening Experiment

For qualitative screening, the treatment time points 7, 5, 3, 2, and 1 day(s) before harvest were accumulated on each single panicle by injecting 5 μL of the ^12^C/^13^C treatment solutions into each of four spikelets per time point, starting with the highest ones and continuing with the next four spikelets below or close beside it. All treatment variants (HT2, T2, and mock) were applied in biological triplicate. Additionally, time point full-ripening was produced as single treatment per condition by treatment of 20 spikelets at flowering stage and harvesting approximately eight weeks later. Within the scope of this experiment, only treated spikelets were further processed and analysed.

#### 4.3.2. Time Course Experiment

Treatment for time course experiment included five time points on separate oat panicles with five biological replicates per treatment and time point. Plants were treated with non-labelled treatment solutions at flowering stage (20 spikelets at once) and harvested immediately (0), 1, 3, or 7 days later and at full-ripening stage (approximately eight weeks after treatment). Treated spikelets, non-treated spikelets, and pooled stem plus side branches were investigated separately to study the formation of toxin derivatives over time and to get an insight into the mobility of both toxins and their major metabolite within the plants.

### 4.4. Sample Preparation

Frozen plant material was milled and extracted with a mixture of acetonitrile/water/formic acid 79/20.9/0.1 (*v*/*v*/*v*) according to [32] prior to the LC-HRMS and LC-HRMS/MS measurements. 

#### 4.4.1. Qualitative Screening Experiment

For qualitative screening, 200 μL portions of the sample extracts (^12^C/^13^C-toxin-treated and mock-treated plants) in solvent acetonitrile/water/formic acid 79/20.9/0.1 (*v*/*v*/*v*) were dried under vacuum at room temperature with CentriVap Refrigerated Concentrator from Labconco (Kansas City, MO, USA) and re-dissolved in 200 μL acetonitrile/water/formic acid 20/80/0.1 (*v*/*v*/*v*). 

#### 4.4.2. Time Course Experiment

Non-labelled extracts for quantitative analysis were measured undiluted or after 400-fold dilution with solvent acetonitrile/water 1/1 (*v*/*v*). Extracts of non-treated plant parts from ^12^C samples (non-treated spikelets and pooled stem plus side branches) were measured undiluted or after 10-fold dilution with acetonitrile/water 1/1 (*v*/*v*). 

### 4.5. Analysis by LC-HRMS and LC-HRMS/MS

#### 4.5.1. Qualitative Screening Experiment

Measurements of ^12^C/^13^C-toxin- and mock-treated samples were performed with an UltiMate 3000 HPLC system combined with an Exactive Plus Orbitrap mass spectrometer (Thermo Fisher Scientific, Bremen, Germany). Chromatographic settings were as follows: column Kinetex C18 (150 × 2.1 mm, 2.6 μm; Phenomenex, Aschaffenburg, Germany); column temperature 25 °C; eluents 0.1% formic acid and 5 mM ammonium formate in water (eluent A) and in methanol (eluent B); flow rate 250 μL/min. Injection volume was set to 10 μL and Gradient method 1 (30 min gradient, from 10% to 100% B plus re-equilibration) was used as described in [32]. Orbitrap measurements were performed in positive and negative electrospray ionisation mode separately with a scan range from *m/z* 130 to 1300. All other mass spectrometric settings were concordant with those of Kluger et al. [25]. Data were acquired and evaluated with Thermo Xcalibur 4.0.27.10 and 2.2 software (both Thermo Fisher Scientific), respectively.

#### 4.5.2. Structure Annotation and Quantification (Time Course Experiment)

For all other measurements a 1290 Infinity UHPLC system combined with a 6550 iFunnel Q-TOF-MS (Agilent Technologies) was used. The chromatographic separation was carried out as recently published for barley [32]. In brief, a Zorbax SB-C18 Rapid Resolution HD column (150 × 2.1 mm, 1.8 μm; Agilent Technologies), a flow rate of 250 μL/min, a column temperature of 30 °C and the same eluents as for Orbitrap measurements (4.5.1) were used. For structure annotation of detected HT2/T2 metabolites, LC-HRMS/MS spectra were acquired using Gradient method 2 (25 min gradient) and undiluted extracts of ^12^C as well as ^12^C/^13^C samples. Time course experiments were carried out with short Gradient method 4 (10 min gradient) and in MS full scan mode. The mass spectrometric settings were similar to Meng-Reiterer et al. [32] with some modifications: Capillary voltage was set to 3000 V, drying gas flow to 16 L/min and sheath gas flow to 11 L/min. Data acquisition and evaluation were made by MassHunter Acquisition software B.06.01 and MassHunter Qualitative Analysis B.07.00 and Quantitative Analysis B.07.01 (Agilent Technologies), respectively.

### 4.6. Metabolite Recognition by MetExtract II (Module TracExtract)

LC-HRMS raw data from qualitative screening experiment were converted to the centroid mode and the mzXML format with ProteoWizard [44]. The subsequent processing with the TracExtract module of the MetExtract II software (in-house programme) [25] enabled the selective recognition of HT2- and T2-derived biotransformation products. To this end, MetExtract II software searched for pairs of corresponding native (M or ^12^C) and partially ^13^C-labelled (M′ or ^13^C) ions of the same metabolite in each MS scan. An intensity ratio of approximately 1/1 (±0.4) and a minimum intensity abundance of 50,000 counts were required for two signals M and M′ to be considered. The *m/z* difference between two corresponding M and M′ signals was proportional to the number of toxin-derived ^13^C-atoms (number of ^13^C-atoms × 1.00335 u). M, M′, and their carbon isotopologs (M + 1 and M′ − 1) had to be present within a mass tolerance of ±4 ppm and a maximum relative intensity abundance error of ±20%. For all corresponding M and M′ ions, EICs were generated (mass tolerance of accurate mass ±5 ppm). Chromatographic peaks present in both EICs were detected with the algorithm of Du et al. [45] as well as examined for coelution (tolerance window of ±10 scans) and similar peak shape (minimum Pearson correlation of 0.85). As the final step, recognised biotransformation product ions were automatically convoluted into feature groups, each of which represents a toxin-derived plant metabolite (minimum Pearson correlation of 0.85).

### 4.7. Annotation of Unknown Metabolites by MetExtract II (Module FragExtract)

FragExtract, developed by Neumann and Lehner et al. [46], was used to confirm the manual interpretation of overlaid LC-HRMS/MS spectra of coeluting M and M′ ions by comparing sum formulas and/or ^12^C/^13^C mass shifts. This module of MetExtract II software automatically detected and annotated fragment ions of successive M and M′ LC-HRMS/MS spectra. Any fragment ion signal present in the LC-HRMS/MS spectra of M was tested for a corresponding fragment ion signal in the LC-HRMS/MS spectra of M′ indicating that these two signals were derived from the analysed biotransformation product. For this, both LC-HRMS/MS spectra were scaled relative to the intensity of their respective precursor signal intensity and only those signals with a minimum scaled relative abundance of 1% were considered for further investigation. The *m/z* difference between two corresponding fragment ion signals was proportional to the number of toxin-derived ^13^C-atoms (number of ^13^C-atoms × 1.00335 u). Zero ^13^C-atoms, which led to no mass shifts of fragment ion signals in M and M′ LC-HRMS/MS spectra, were allowed to account for fragments originating from native conjugated molecules carrying no ^13^C-atoms. If two corresponding fragment ion signals showed a maximum mass tolerance of ±30 ppm and a maximum relative intensity abundance error of ±40%, they were annotated with the number of carbon atoms originating from the labelled tracer compound. Other signals from both LC-HRMS/MS spectra not successfully matching were discarded as noise. For matched fragment signal pairs, putative sum formulas were generated with the Seven Golden Rules [47]. These sum formulas had to have at least as many carbon atoms as their fragment ion signals were annotated with.

### 4.8. Quantification Experiments

LC-Q-TOF (4.5.2) with Gradient method 4 and full scan mode was used for all quantitative measurements. For absolute quantification of HT2, T2, and HT2-3-*O*-*β*-d-Glc in extracts of ^12^C-HT2- and ^12^C-T2-treated oat spikelets, samples were diluted 400-fold with acetonitrile/water 1/1 (*v*/*v*). Standard mixtures in the same solvent with concentrations at five levels between 3–300 μg/L were prepared (mostly, four of them were used) to perform external linear calibration (1/x weighted). Sodium adducts of the respective analytes were extracted (exact mass ±20 ppm) and integrated when EICs were above the limit of quantification (LOQ, signal-to-noise ratio of 10 in matrix). Amounts of HT2, T2, and HT2-3-*O*-*β*-d-Glc were calculated in μmol/treated part and corrected by matrix effects obtained for the respective metabolite. The matrix effects were determined with mock samples harvested at 1 day and full-ripening, separately, in biological triplicate. To this end, 50 μL of a 200 μg/L (per analyte) stock solution including HT2, T2, and HT2-3-*O*-*β*-d-Glc were evaporated and dissolved in 200 μL of 400-fold diluted matrix to obtain 50 μg/L (per analyte). The EIC peak areas of matrix-affected sodium adducts were then divided by the respective areas of a 50 μg/L standard mix and multiplied by 100.

All other metabolites were measured in undiluted extracts of ^12^C-HT2- and ^12^C-T2-treated oat spikelets by generating EICs of *m/z* traces corresponding to ammonium adducts (exact mass ±20 ppm). Metabolite peak areas were normalised by the respective weight of the treated plant part. 

Semi-quantitative estimation of HT2, T2, and HT2-3-*O*-*β*-d-Glc in non-treated plant parts (non-treated spikelets and pooled stem plus side branches) of the ^12^C samples was based on undiluted or 10-fold diluted extracts, linear calibration (1/x weighted) in the range of 10–1000 μg/L, and EICs of ammonium adducts (exact mass ±25 ppm), which are less prone to matrix effects compared to the corresponding sodium adducts. Since the measured concentrations of HT2-3-*O*-*β*-d-Glc were partially out of the calibration range (up to five times higher than the highest calibrant), linear calibration was extrapolated. Due to this procedure and no corrections of matrix effects, the indicated amounts in μmol/non-treated part represent semi-quantitative estimations.

## Figures and Tables

**Table 1:** List of identified and annotated metabolites of HT-2 toxin in oats.

ID	Metabolite	Retention Time (min)	Elemental Composition ^a^	Accurate Mass ^b^	Adduct ^b^	n_c_^c^	Mass Error (ppm)
	HT2 *	15.69	C_22_H_32_O_8_	442.2446	[M+NH_4_]^+^	22	2.4
1	15-Acetyl-T2-tetraol-Glc **	5.45	C_23_H_34_O_12_	520.2408	[M+NH_4_]^+^	17	3.7
2	Dehydro-15-acetyl-T2-tetraol-Glc ^d,^***	8.09	C_23_H_32_O_12_	518.2250	[M+NH_4_]^+^	17	3.5
3	Hydroxy-HT2-HexGlc **	10.21	C_34_H_52_O_19_	782.3468	[M+NH_4_]^+^	22	3.4
4	Hydroxy-HT2-HexGlc **	10.88	C_34_H_52_O_19_	809.3100	[M+HCOO]^−^	22	1.9
5	Hydroxy-HT2-Glc **	11.08	C_28_H_42_O_14_	620.2927	[M+NH_4_]^+^	22	2.3
6	Hydroxy-HT2 **	11.71	C_22_H_32_O_9_	458.2398	[M+NH_4_]^+^	22	2.9
7	Hydroxy-HT2-anhydro-HexGlc **	12.37	C_34_H_50_O_18_	764.3355	[M+NH_4_]^+^	22	2.6
8	T2-triol-Glc **	13.81	C_26_H_40_O_12_	589.2508	[M+HCOO]^−^	20	1.1
9	Dehydro-HT2-Glc **	14.05	C_28_H_40_O_13_	629.2458	[M+HCOO]^−^	22	1.1
10	HT2-HexGlc **	14.23	C_34_H_52_O_18_	766.3509	[M+NH_4_]^+^	22	2.2
11	HT2-HexGlc **	14.76	C_34_H_52_O_18_	766.3511	[M+NH_4_]^+^	22	2.5
12	HT2-malylGlc **	14.85	C_32_H_46_O_17_	720.3087	[M+NH_4_]^+^	22	1.9
13	HT2-3-*O*-*β*-d-Glc *	15.00	C_28_H_42_O_13_	604.2973	[M+NH_4_]^+^	22	1.5
14	HT2-MalGlc ^d,^**	15.11	C_31_H_44_O_16_	690.2994	[M+NH_4_]^+^	22	3.8
15	HT2-anhydro-HexGlc **	15.51	C_34_H_50_O_17_	748.3404	[M+NH_4_]^+^	22	2.4
16	T2 ^d,^*	16.87	C_24_H_34_O_9_	484.2549	[M+NH_4_]^+^	22	1.6

HT2, HT-2 toxin; T2, T-2 toxin; Glc, glucoside; MalGlc, malonylglucoside; HexGlc, hexosylglucoside; ^a^ Elemental composition of uncharged metabolite; ^b^ Accurate mass (mean *m/z*) of the most abundant adduct of MetExtract II derived features; ^c^ Number of C-atoms originating from parent toxin; ^d^ Detected by targeted search for barley- [32] and wheat- [33] derived metabolites; * Structure confirmation with standard by comparison of retention time, accurate mass and HRMS/MS-spectrum; ** Structure annotation with accurate mass, number of parent toxin-derived C-atoms, HRMS/MS-spectrum, and by assessment of retention time; *** Structure annotation with accurate mass, number of parent toxin-derived C-atoms, and by assessment of retention time.

**Table 2:** List of identified and annotated metabolites of T-2 toxin in oats.

ID	Metabolite	Retention Time (min)	Elemental Composition ^a^	Accurate Mass ^b^	Adduct ^b^	n_c_^c^	Mass Error (ppm)
	T2 *	16.83	C_24_H_34_O_9_	484.2548	[M+NH_4_]^+^	24	1.4
1	15-Acetyl-T2-tetraol-Glc ^d,^**	5.46	C_23_H_34_O_12_	520.2407	[M+NH_4_]^+^	17	3.6
2	Dehydro-15-acetyl-T2-tetraol-Glc ^d,^***	8.10	C_23_H_32_O_12_	518.2248	[M+NH_4_]^+^	17	3.1
3	Hydroxy-HT2-HexGlc ^d,^**	10.23	C_34_H_52_O_19_	782.3462	[M+NH_4_]^+^	22	2.7
5	Hydroxy-HT2-Glc **	11.08	C_28_H_42_O_14_	620.2926	[M+NH_4_]^+^	22	2.1
6	Hydroxy-HT2 **	11.71	C_22_H_32_O_9_	458.2396	[M+NH_4_]^+^	22	2.5
7	Hydroxy-HT2-anhydro-HexGlc ^d,^**	12.36	C_34_H_50_O_18_	764.3348	[M+NH_4_]^+^	22	1.7
8	T2-triol-Glc **	13.81	C_26_H_40_O_12_	589.2508	[M+HCOO]^−^	20	1.1
9	Dehydro-HT2-Glc **	14.04	C_28_H_40_O_13_	629.2458	[M+HCOO]^−^	22	1.1
10	HT2-HexGlc **	14.23	C_34_H_52_O_18_	766.3509	[M+NH_4_]^+^	22	2.2
11	HT2-HexGlc **	14.78	C_34_H_52_O_18_	766.3511	[M+NH_4_]^+^	22	2.5
12	HT2-malylGlc **	14.84	C_32_H_46_O_17_	720.3088	[M+NH_4_]^+^	22	2.0
13	HT2-3-*O*-*β*-d-Glc *	14.99	C_28_H_42_O_13_	604.2975	[M+NH_4_]^+^	22	1.9
14	HT2-MalGlc ^d,^**	15.11	C_31_H_44_O_16_	690.2990	[M+NH_4_]^+^	22	3.2
15	HT2-anhydro-HexGlc **	15.50	C_34_H_50_O_17_	729.2983	[M−H]^−^	22	1.1
17	HT2 *	15.68	C_22_H_32_O_8_	442.2446	[M+NH_4_]^+^	22	2.4
18	3-Acetyl-T2 *	18.07	C_26_H_36_O_10_	526.2653	[M+NH_4_]^+^	24	1.2
19	Feruloyl-T2 ^d,^**	19.17	C_34_H_42_O_12_	660.3022	[M+NH_4_]^+^	24	1.1

HT2, HT-2 toxin; T2, T-2 toxin; Glc, glucoside; MalGlc, malonylglucoside; HexGlc, hexosylglucoside; ^a^ Elemental composition of uncharged metabolite; ^b^ Accurate mass (mean *m/z*) of the most abundant adduct of MetExtract II derived features; ^c^ Number of C-atoms originating from parent toxin; ^d^ Detected by targeted search for HT2 metabolites known to be produced in oats or for barley- [32] and wheat- [33] derived metabolites; * Structure confirmation with standard by comparison of retention time, accurate mass, and HRMS/MS-spectrum; ** Structure annotation with accurate mass, number of parent toxin-derived C-atoms, HRMS/MS-spectrum, and by assessment of retention time; *** Structure annotation with accurate mass, number of parent toxin-derived C-atoms, and by assessment of retention time.

**Table 3:** Mobility of HT2-3-*O*-*β*-d-Glc in HT2-treated oat panicles.

Time Points (Days)	% of HT2 at 0 Days ^a^ Stem plus Side Branches	% of HT2 at 0 Days Non-Treated Spikelets
0	0.0 ± 0.0	<LOD
1	4.4 ± 2.6	<LOD
3	3.0 ± 2.6	<LOD
7	2.4 ± 2.2	<LOD
full-ripening	1.2 ± 1.2	<LOD

HT2, HT-2 toxin; Glc, glucoside; LOD, limit of detection was estimated to correspond to the signal-to-noise ratio of three in matrix; Oat panicles were treated with 200 μg non-labelled HT2 (for each time point toxins were applied on separate panicles in five biological replicates), harvested directly (zero), and one, three, or seven days after treatment or at the full-ripening stage. For non-treated plant parts (non-treated spikelets and pooled stem plus side branches), semi-quantitative analysis was performed (^a^ percent mean value ± standard deviation in percentage points is stated).

**Table 4:** Mobility of HT2-3-*O*-*β*-d-Glc in T2-treated oat panicles.

Time Points (Days)	% of T2 + HT2 at 0 Days ^a^ Stem plus Side Branches	% of T2 + HT2 at 0 Days Non-Treated Spikelets
0	0.0 ± 0.0	<LOD
1	1.3 ± 0.9	<LOD
3	0.7 ± 0.3	<LOD
7	1.0 ± 0.8	<LOD
full-ripening	0.3 ± 0.3	<LOD

HT2, HT-2 toxin; T2, T-2 toxin; Glc, glucoside; LOD, limit of detection was estimated to correspond to the signal-to-noise ratio of three in matrix; Oat panicles were treated with 200 μg non-labelled T2 (for each time point toxins were applied on separate panicles in five biological replicates), harvested directly (zero), and one, three, or seven days after treatment or at the full-ripening stage. For non-treated plant parts (non-treated spikelets and pooled stem plus side branches), semi-quantitative analysis was performed (^a^ percent mean value ± standard deviation in percentage points is stated).

## Supplementary Materials

The following are available online at www.mdpi.com/2072-6651/8/12/364/s1, Figure S1: LC-HRMS/MS-spectrum and putative structure formula of the HT2/T2 plant metabolite T2-triol-Glc (8). Formate adduct (marked with a diamond) was fragmented with a collision energy of 20 eV on a LC-Q-TOF-system. Characteristic fragments are highlighted. HT2, HT-2 toxin; T2, T-2 toxin; Glc, glucoside; isoval acid, isovaleric acid, Figure S2: LC-HRMS/MS-spectrum and putative structure formula of the HT2/T2 plant metabolite Hydroxy-HT2-HexGlc (3). Ammonium adduct (marked with a diamond) was fragmented with a collision energy of 12 eV on a LC-Q-TOF-system. Characteristic fragments are highlighted. Additional hydroxyl group was confirmed to be located at isovaleric acid moiety. * Sum formula and/or ^12^C/^13^C mass shift confirmed by FragExtract module of the MetExtract II software. HT2, HT-2 toxin; T2, T-2 toxin; HexGlc, hexosylglucoside; isoval acid, isovaleric acid, Figure S3: LC-HRMS/MS-spectrum and putative structure formula of the HT2/T2 plant metabolite Hydroxy-HT2 (6). Ammonium adduct (marked with a diamond) was fragmented with a collision energy of 8 eV on a LC-Q-TOF-system. Characteristic fragments are highlighted. Additional hydroxyl group was confirmed to be located at isovaleric acid moiety. HT2, HT-2 toxin; T2, T-2 toxin; isoval acid, isovaleric acid. Figure S4: LC-HRMS/MS-spectrum and putative structure formula of the HT2/T2 plant metabolite HT2-malylGlc (12). Ammonium adduct (marked with a diamond) was fragmented with a collision energy of 13 eV on a LC-Q-TOF-system. Characteristic fragments are highlighted. * Sum formula and/or ^12^C/^13^C mass shift confirmed by FragExtract module of the MetExtract II software. HT2, HT-2 toxin; T2, T-2 toxin; Glc, glucoside; isoval acid, isovaleric acid, Figure S5: LC-HRMS/MS-spectrum of the HT2/T2 plant metabolite Hydroxy-HT2-anhydro-HexGlc (7). Ammonium adduct (marked with a diamond) was fragmented with a collision energy of 15 eV on a LC-Q-TOF-system. Characteristic fragments are highlighted. Additional hydroxyl group was confirmed to be located at isovaleric acid moiety. * Sum formula and/or ^12^C/^13^C mass shift confirmed by FragExtract module of the MetExtract II software. HT2, HT-2 toxin; T2, T-2 toxin; HexGlc, hexosylglucoside; isoval acid, isovaleric acid, Figure S6: LC-HRMS/MS-spectrum of the HT2/T2 plant metabolite HT2-anhydro-HexGlc (15). Ammonium adduct (marked with a diamond) was fragmented with a collision energy of 12 eV on a LC-Q-TOF-system. Characteristic fragments are highlighted. * Sum formula and/or ^12^C/^13^C mass shift confirmed by FragExtract module of the MetExtract II software. HT2, HT-2 toxin; T2, T-2 toxin; HexGlc, hexosylglucoside; isoval acid, isovaleric acid.
