# Supplementary material for: Metabolism of HT-2 Toxin and T-2 Toxin in Oats
Source: Toxins (Basel). 2016 Dec 5;8(12):364. doi: 10.3390/toxins8120364 (PMC5198558; doi:10.3390/toxins8120364)
Supplement: Supplementary file 1 [file toxins-08-00364-s001.pdf]

Supplementary Materials: Metabolism of HT-2 Toxin and T-2 Toxin in Oats

Jacqueline Meng-Reiterer, Christoph Bueschl, Justyna Rechthaler, Franz Berthiller, Marc Lemmens and Rainer Schuhmacher

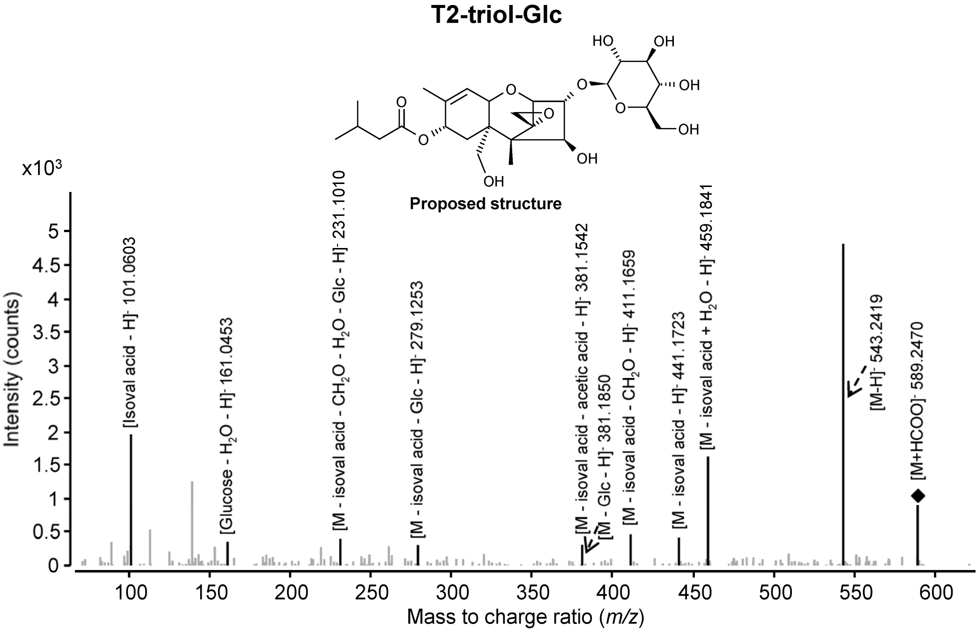

Figure S1. LC-HRMS/MS-spectrum and putative structure formula of the HT2/T2 plant metabolite T2-triol-Glc (8). Formate adduct (marked with a diamond) was fragmented with a collision energy of 20 eV on a LC-Q-TOF-system. Characteristic fragments are highlighted. HT2, HT-2 toxin; T2, T-2 toxin; Glc, glucoside; isoval acid, isovaleric acid.

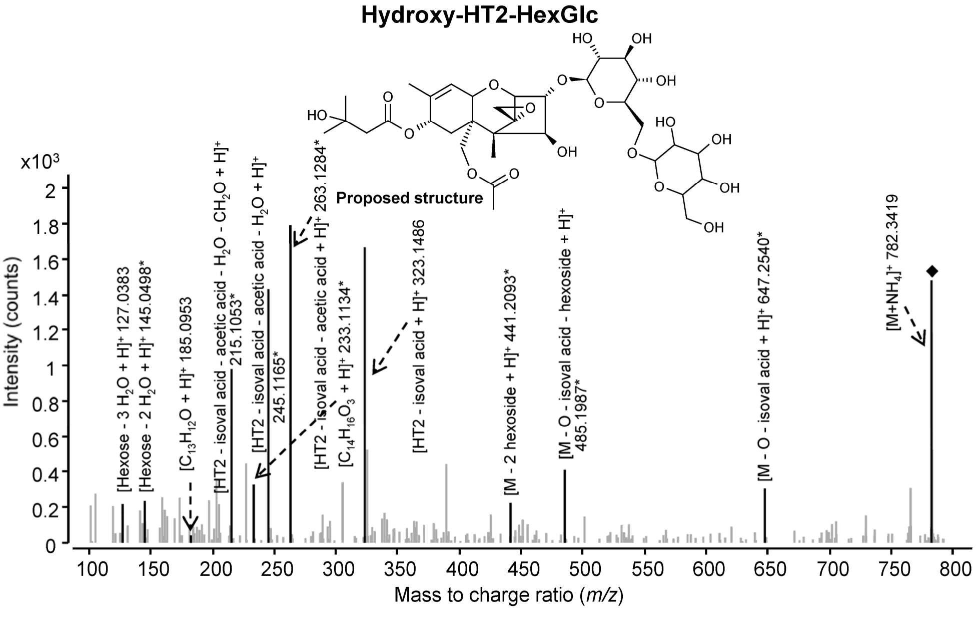

Figure S2. LC-HRMS/MS-spectrum and putative structure formula of the HT2/T2 plant metabolite Hydroxy-HT2-HexGlc (3). Ammonium adduct (marked with a diamond) was fragmented with a collision energy of 12 eV on a LC-Q-TOF-system. Characteristic fragments are highlighted. Additional hydroxyl group was confirmed to be located at isovaleric acid moiety. * Sum formula and/or $^{12}C/^{13}C$ mass shift confirmed by FragExtract module of the MetExtract II software. HT2, HT-2 toxin; T2, T-2 toxin; HexGlc, hexosylglucoside; isoval acid, isovaleric acid.

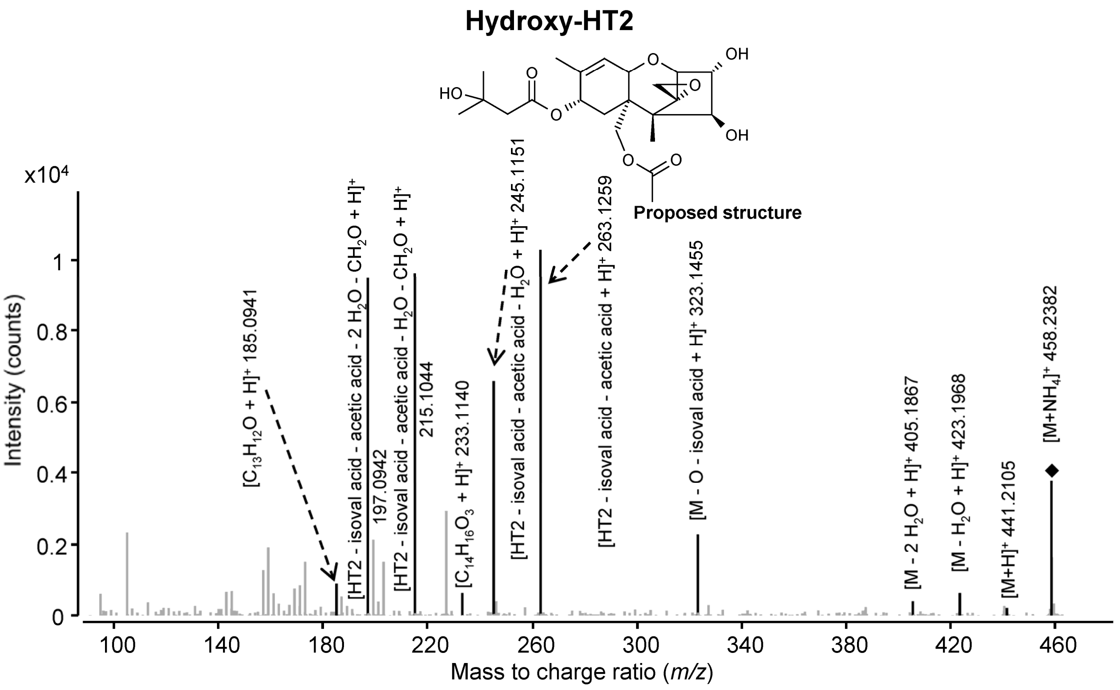

Figure S3. LC-HRMS/MS-spectrum and putative structure formula of the HT2/T2 plant metabolite Hydroxy-HT2 (6). Ammonium adduct (marked with a diamond) was fragmented with a collision energy of 8 eV on a LC-Q-TOF-system. Characteristic fragments are highlighted. Additional hydroxyl group was confirmed to be located at isovaleric acid moiety. HT2, HT-2 toxin; T2, T-2 toxin; isoval acid, isovaleric acid.

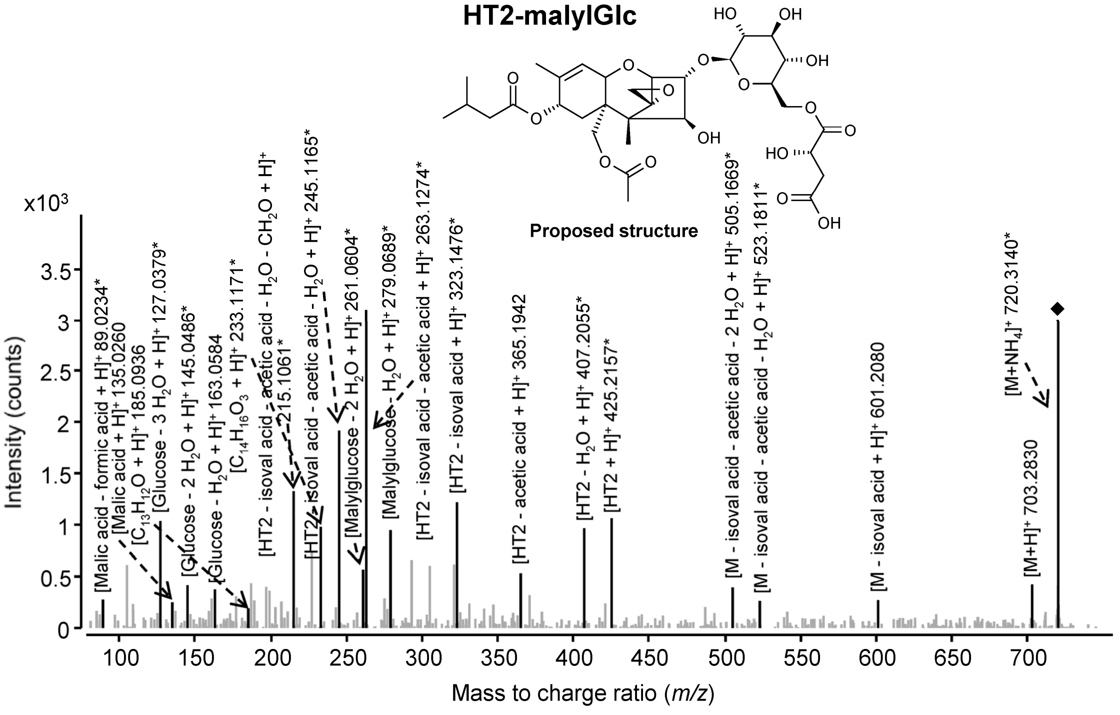

Figure S4. LC-HRMS/MS-spectrum and putative structure formula of the HT2/T2 plant metabolite HT2-malylGlc (12). Ammonium adduct (marked with a diamond) was fragmented with a collision energy of 13 eV on a LC-Q-TOF-system. Characteristic fragments are highlighted. * Sum formula and/or $^{12}C/^{13}C$ mass shift confirmed by FragExtract module of the MetExtract II software. HT2, HT-2 toxin; T2, T-2 toxin; Glc, glucoside; isoval acid, isovaleric acid.

Hydroxy-HT2-anhydro-HexGlc

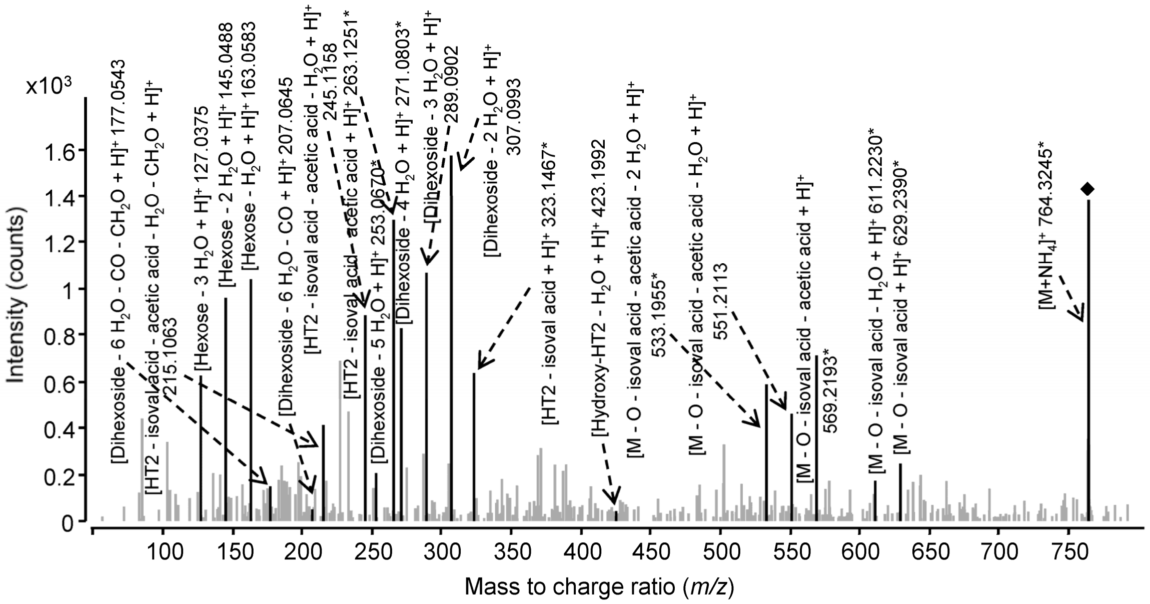

Figure S5. LC-HRMS/MS-spectrum of the HT2/T2 plant metabolite Hydroxy-HT2-anhydro-HexGlc (7). Ammonium adduct (marked with a diamond) was fragmented with a collision energy of 15 eV on a LC-Q-TOF-system. Characteristic fragments are highlighted. Additional hydroxyl group was confirmed to be located at isovaleric acid moiety. * Sum formula and/or $^{12}C/^{13}C$ mass shift confirmed by FragExtract module of the MetExtract II software. HT2, HT-2 toxin; T2, T-2 toxin; HexGlc, hexosylglucoside; isoval acid, isovaleric acid.

HT2-anhydro-HexGlc

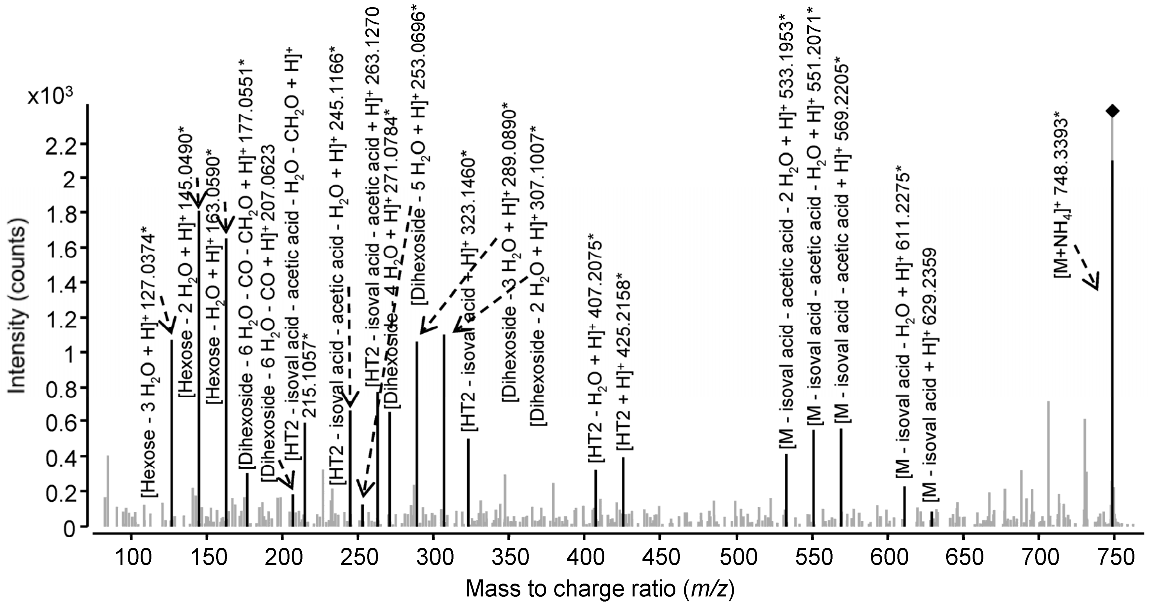

Figure S6. LC-HRMS/MS-spectrum of the HT2/T2 plant metabolite HT2-anhydro-HexGlc (15). Ammonium adduct (marked with a diamond) was fragmented with a collision energy of 12 eV on a LC-Q-TOF-system. Characteristic fragments are highlighted. * Sum formula and/or $^{12}C/^{13}C$ mass shift confirmed by FragExtract module of the MetExtract II software. HT2, HT-2 toxin; T2, T-2 toxin; HexGlc, hexosylglucoside; isoval acid, isovaleric acid.
